# Mitogenomes revealed the history of bison colonization of Northern Plains after the Last Glacial Maximum

**DOI:** 10.1038/s41598-023-37599-8

**Published:** 2023-07-14

**Authors:** Igor V. Ovchinnikov, Blake McCann

**Affiliations:** 1 Department of Biology, University of North Dakota, Grand Forks, ND USA; 2 Theodore Roosevelt National Park, Medora, ND USA

**Keywords:** Evolutionary genetics, Evolutionary biology

## Abstract

American bison demonstrated differential patterns of extinction, survival, and expansion since the terminal Pleistocene. We determined population dynamics of the Northern Great Plains bison using 40 mitochondrial genomes from radiocarbon dated remains with the age ranging from 12,226 to 167 calibrated years before present. Population dynamics correlated with environmental and anthropogenic factors and was characterized by three primary periods: terminal Pleistocene population growth starting 14,000 years ago, mid Holocene demographic stability between 6700 and 2700 years ago, and late Holocene population decline in the last 2700 years. Most diversification of mtDNA haplotypes occurred in the early Holocene when bison colonized new territories opened by retreating ice sheets. Holocene mtDNA lineages were not found in modern bison and lacked association with archaeological sites and morphological forms.

## Introduction

Bison are the largest megafaunal taxa in North America to survive the terminus of the late Pleistocene Epoch. Based on reliable paleontological and genetic evidence, Beringian steppe bison (*Bison priscus*) entered North America from Asia through the Bering Land Bridge in the late Middle Pleistocene approximately 300,000–135,000 years ago (ya)^1,2^. The origin of long-horn bison (*B. latifrons*), another early North American species, remains open. According to modern understanding, *B. latifrons* evolved from *B. priscus* in the central part of North America before 120,000 ya^3,4^. *B. antiquus* appeared in the paleontological record around 60,000 ya and in the Late Pleistocene this species occupied the territory from Alaska to the Mexican highlands and Florida^5^. Adaptation of this species to climatic and environmental fluctuations led to morphological changes and the appearance of *B. bison* through the transitional morphospecies of *B. occidentalis*^6,7^.

Across late Pleistocene geographic distribution, bison demonstrated differential patterns of local extirpation and survival. Similar to other large mammals that went extinct in North America, steppe bison (*Bison priscus*) disappeared in Alaskan Beringia between 14,000 and 10,000 ya^8,9^ due to elevated climate changes during that time affecting environmental factors such as ambient temperature, rangeland moisture, and vegetation^10,11^. In contrast, in the Great Plains, bison successfully adapted to drastic climatic fluctuations and ecological changes occurring since the Last Glacial Maximum (LGM) 26,500–19,000 ya^12^. In the early Holocene, bison became established as a dominant species on the Great Plains, flourishing during this Epoch despite wide climatic variability, with tens of millions roaming the prairies before their anthropogenic decimation in the nineteenth century^13^.

The different fates of bison across their late Pleistocene wide geographic distribution suggests variable response to environmental factors that resulted in local extirpation of bison in the Arctic and survival in other parts of North America, ranging from Interior Alaska to northern Mexico^14^. The consequences of environmental changes can be documented through the study of evolutionary demography of geographic populations^15^. However, current knowledge about past bison population dynamics is limited to several publications focusing on the demography of Beringian steppe bison^1,10,16^, dispersal through the ice-free corridor between Laurentide and Cordilleran ice sheets^17^, and temporal periods of bison migration from western (northeast Asia) to eastern (Alaska and Yukon) Beringia^4^.

The demography of Beringian steppe bison was originally described as a boom-bust model determined using a section of mtDNA control region^1^. Bison indicated exponential population growth upon entering Alaskan Beringia across the Bering Land Bridge 300,00–130,000 ya, reaching a population maximum approximately 37,000 ya, followed by a continuous decline with a 230 times reduction of effective population size (N_e_) prior to the nineteenth century anthropogenic bottleneck^1^. That decline was linked to environmental changes, but the specific alterations preceding the dramatic population decline were not fully described. Another study, based on a subset of steppe bison mtDNA sequences, demonstrated a conflicting scenario, with population growth from 50,000 to 35,000 ya, followed by climate-caused decline in N_e_ that reached its five-fold minimum 10,000–11,000 ya. Smaller population growth was detected after 10,000 ya, reaching a population maximum 5000 ya^10^.

More sensitive demographic analysis of the same dataset^1^ based on the Bayesian skyline method revealed that between 70,000 and 25,000 ya the population size was high and stable followed by a later population decline that began 25,000 ya in the beginning of the LGM and reached a N_e_ minimum approximately 10,000 ya, at a time when many megafaunal taxa went extinct throughout North America^16^. The latter timepoint (25,000 ya) for population decline was further supported with recent data indicating that *B. priscus* was abundant on Alaska’s North Slope 45,000–35,000 ya^18^. That study further supports that population growth then occurred from the early Holocene approximately 10,000 ya through the mid Holocene to as recently as 5000 ya, when the population reached its Holocene N_e_ maximum. This maximum was substantially less than the population peak before the LGM and was followed by a small population decline. The reasons for bison population reduction in the late Holocene were not clearly explained except that humans could have played a role in the decline^16^.

However, conclusions based on bison in Beringia should not be extended to contemporaneous bison populations elsewhere in North America, because of ecological plasticity and genetic discontinuity of the species across continental scales^1,4,19^. Further, both studies^1,10^ were based on short mtDNA sequences, with limited samples from the Holocene analyzed in context of late Pleistocene Beringian specimens from Siberia and Alaska.

Based on paleontological and archaeological records, bison population sizes in the Holocene varied across their distributional range including the Great Plains^20^. However, exact demography as the dynamics of effective population size in local populations in the past remains unknown but can be reasonably documented based on ancient mitogenome sequences available from throughout their spatial range. Bayesian demographic analyses are useful in identifying timing of changes of bison populations which can then be associated with other drivers of environmental events. To date, only 31 complete or near complete mitochondrial genome sequences from steppe bison (*B. priscus*) were described from eastern Beringia and the migration corridor between Cordilleran and Laurentide ice sheets^4,21,22^. Only four of them came from Holocene specimens, including one from Charlie Lake Cave (British Columbia; KX269118), two from Clover Bar Sand and Gravel (Alberta; KX269120, KX269143) and one from Whitehorse (Yukon, MF134653). No mitogenome sequences, however, are available from the expansive territory of the Holocene range, including most of the Great Plains.

The Great Plains represents the largest biome in North America with diverse climatic factors and vegetation across prairie systems^13^. To minimize effects of differential ecology on observed genetic variation, we focused on Holocene bison specimens with determined radiocarbon dates^19^ from a relatively small area of the Northern Great Plains (NGP)^23^. In this study, we tested the hypothesis whether Northern Plains bison demonstrated distinct population dynamics from Beringian bison^1,10,16^, using sequences representing a global bison population containing late Pleistocene, Holocene and modern mitogenomes. To test this hypothesis, we collected and reconstructed complete mitochondrial genomes from different Holocene periods to evaluate the mostly unknown genetic variation of Holocene bison. We also tested the time of origins of mtDNA haplotypes, their survival through the Holocene, and their relationship with *B. priscus* and modern mtDNA lineages that survived the anthropogenic bottleneck in the nineteenth century. Finally, we estimated N_e_ for bison roaming the NGP throughout the Holocene, including pre-extirpation N_e_ and compared the N_e_ values with N_e_ in the global population.

## Results

### Phylogenetic analysis of mitochondrial genomes

**Figure 1:**
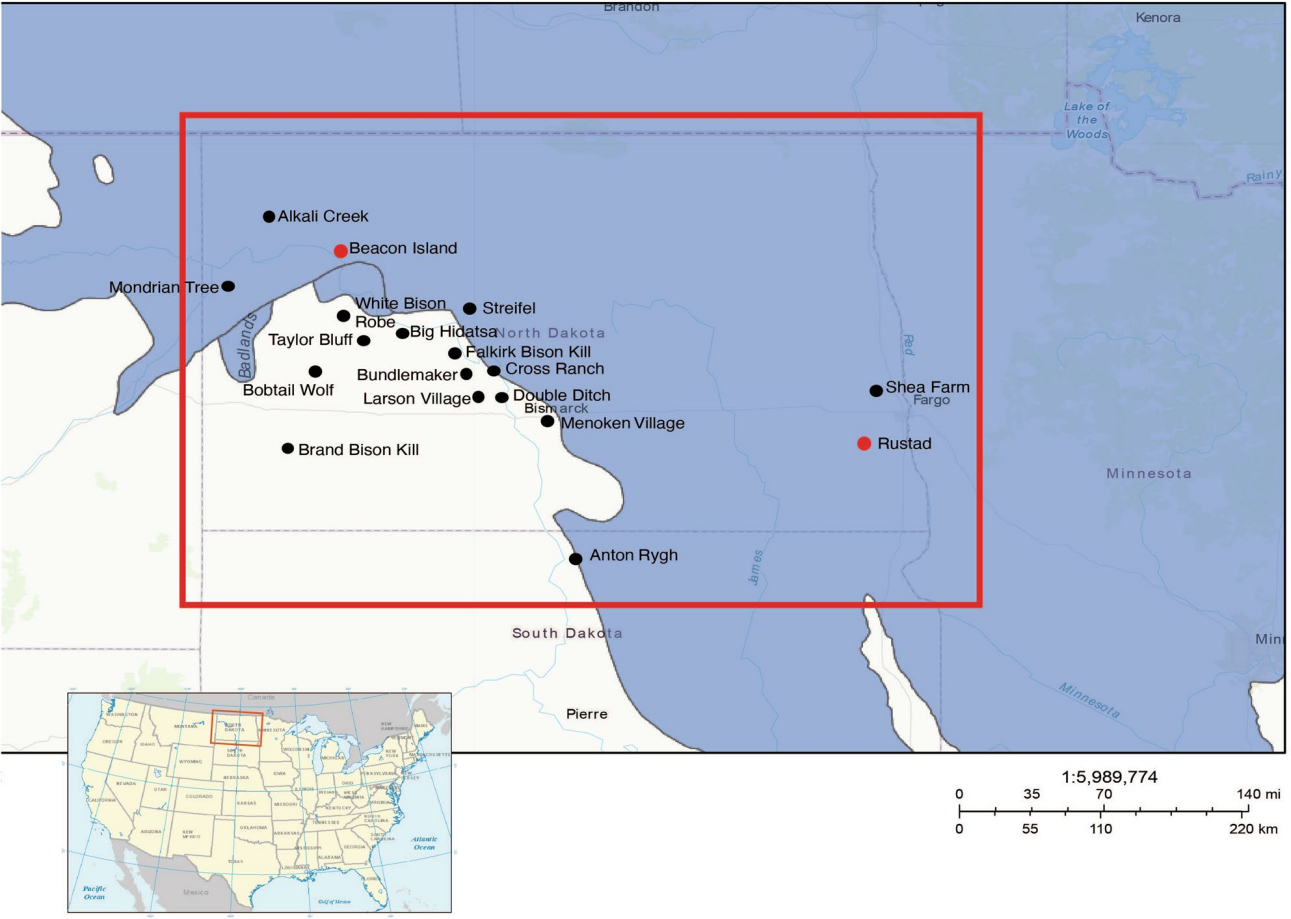
Archaeological sites. Archaeological sites in the NGP (North Dakota and northern South Dakota) where bison specimens were collected are shown within a red rectangle. The blue area shows the Laurentide ice sheet extent after the Last Glacial Maximum from about 18,000 ya (https://www.arcgis.com/home/item.html?id=f1e7378b962d42168fdefec3b6eb8b5f) to 12,300 ya^26^. The small, inserted rectangle indicates the location of the sample area in the U.S. The U.S. map was created using the USA traditional layout in ArcMap (ArcGIS 10.8.2 by Esri). Red dots show the sites with *B. antiquus* remains. Black dots show the sites with *B. bison* remains.

We carried out the target enrichment of DNA libraries prepared from 47 bison bone and tooth specimens for bison mitochondrial genome sequences followed by high throughput sequencing. Forty specimens from 18 archaeological sites (Fig. 1; Supplementary Figs. S1, S2) yielded complete mitochondrial genome sequences (Supplementary Data S1, S2; Supplementary Figs. S3, S4). Based on morphological characteristics, bison specimens from Beacon Island (32MN234) and Rustad (32R1775) belonged to *B. antiquus*^7,24^. The bison remains from other sites were identified as *B. bison*^25^.

**Figure 2:**
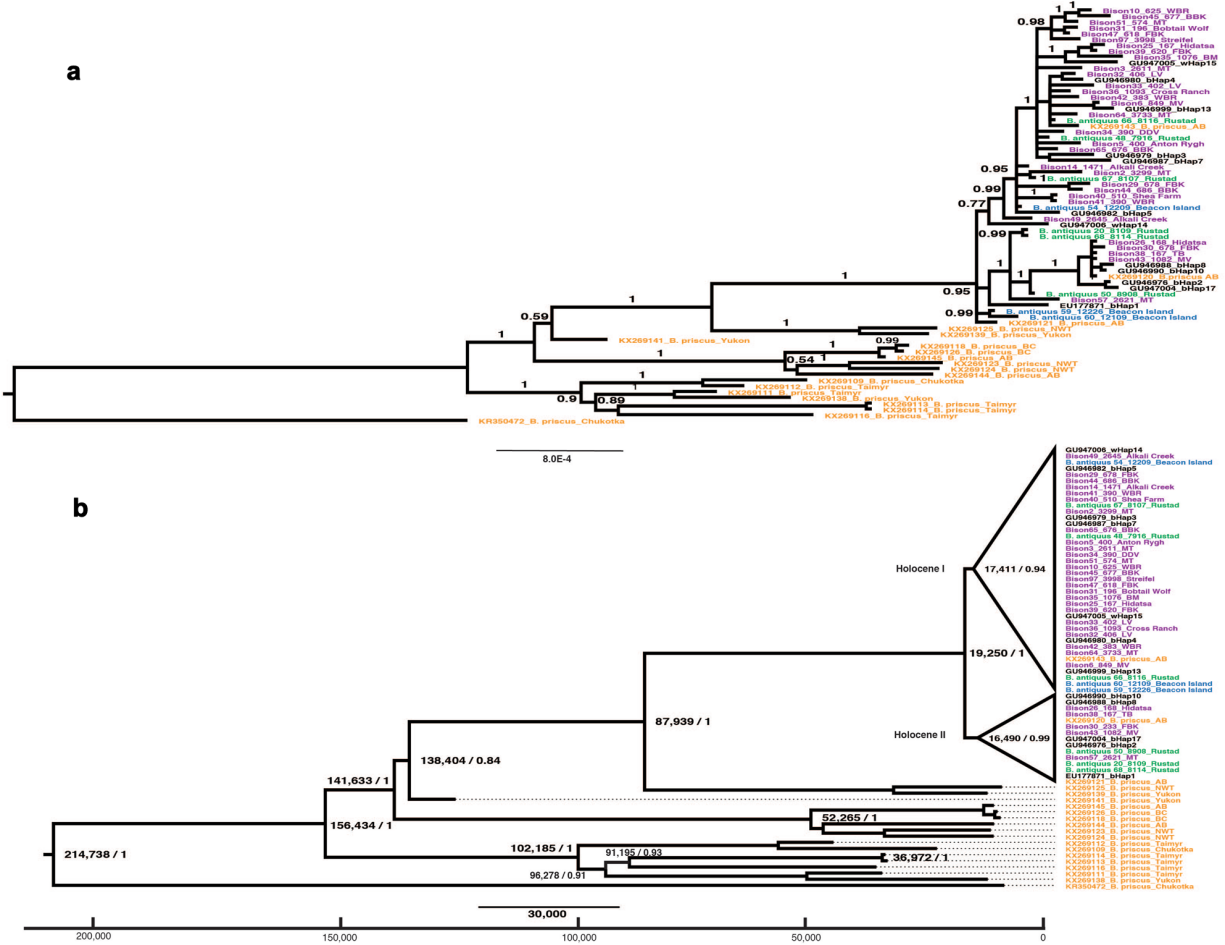
Phylogenetic trees of bison mtDNA. (**a**) Uncalibrated phylogenetic tree of bison mitochondrial genome sequences. Node numbers indicate posterior probability values. The scale shows substitutions per nucleotide. (**b**) Bayesian phylogenetic tree of mitochondrial genome sequences calibrated according to age of bison remains. Node numbers refer to calibrated years before present and posterior probability values. The scale corresponds to 30,000 calibrated years before present. The x axis indicates calibrated years before present. Both trees include mtDNA sequences of 40 Holocene bison from the NGP obtained in this study, 20 *B. priscus*^4,27^, and 12 bison representing the primary modern mtDNA haplotypes^28^. Different groups of bison samples are labeled by the following colors: purple for the Holocene *B. bison* (this study), green for *B. antiquus* from Rustad (this study), blue for *B. antiquus* from Beacon Island (this study), orange for *B. priscus*^4,27^, and black for modern bison^28^. Site code: *BBK* Brand Bison Kill, *BM* Bundlemaker, *DDV* Double Ditch Village, *FBK* Falkirk Bison Kill, *Hidatsa* Big Hidatsa, *LV* Larson Village, *MT* Mondrian Tree, *MV* Menoken Village, *TB* Taylor Bluff, *WBR* White Buffalo Robe.

We performed Bayesian phylogenetic and Bayesian skyline analyses with multiple sets of complete mitochondrial genomes. First, we built an uncalibrated phylogenetic tree for a bison global population using available mitogenome datasets, consisting of our 40 mitogenomes from Holocene *B. bison* and *B. antiquus* previously radiocarbon dated^19^, 20 mitogenomes of *B. priscus* of the best quality without stretches of undetermined nucleotides from radiocarbon dated remains originating from eastern and western Beringia^4,27^, and 12 modern *B. bison* representing the currently existing modern mtDNA haplotypes^28^. The phylogenetic analysis indicated strong statistical support for separating a group consisting of Holocene and modern bison and three haplotypes (KX269120, KX269121, and KX269143) from *B. priscus* remains excavated in Clover Bar Sand and Gravel site in Alberta and corresponding to Clade 1A in^22^. The sister clade to the above group was composed of two sequences from Alaskan Beringia dated to the terminal Pleistocene. These two mtDNA sequences were isolated from *B. priscus* specimens from Inuvik, Northwest Territories (KX269125) and Old Crow, Yukon (KX269139) and dated to the time when the corridor between Laurentide and Cordilleran ice sheets opened for migrations after the ice sheets that covered the area 23,000–13,400 ya retreated^17^ (Fig. 2A).

Next, we used radiocarbon dates to calibrate the Bayesian phylogenetic tree for a late Pleistocene-Holocene-modern set (Fig. 2B) and determined that the most recent common ancestor of the mtDNA lineages of Beringian and North American bison lived 214,738 ya (95% confidence interval of 182,812–249,260 ya), with clear separation of western and eastern Beringian lineages 156,434 ya (143,308–171,378 ya). This late date is close to the time of a common maternal ancestor of North American bison living 135,000–195,000 ya^4^. All Holocene, modern and three *B. priscus* sequences from Alberta clustered together and separated 87,939 ya (73,498–103,417 ya) from a group composed of two mtDNA sequences from *B. priscus* on the north of the ice-free corridor and in turn became separated 34,539 ya (25,395–44,793 ya).

The radiation of Holocene, modern and latest *B. priscus* lineages happened 19,250 ya (15,383–24,281 ya), which correlates with the end of the LGM. It also coincides with the temporal range determined as the time of a common ancestor (15,000–23,000 ya) for fossil bison that lived south of 60° N and modern *B. bison* determined by the mtDNA control region^1,17^. This was a period when the corridor between the Laurentide and Cordilleran ice sheets was closed to migrations^17^, eliminating gene flow between the Beringian and continental populations. This group diverged into two clades denoted here as Holocene I and Holocene II with the time of diversification of 17,411 (14,372–21,500 ya) and 16,490 ya (13,736–20,402 ya), respectively (Fig. 2B). Both clades included mtDNA sequences from *B. priscus*, Holocene mitogenomes, and modern mtDNA sequences. Sequences from *B. antiquus* from three Beacon Island samples (B54, B59, and B60) with the temporal range of 12,109–12,226 ya were only included in the basal positions in Clade Holocene I. Sequences from Rustad, Mondrian Tree, Big Hidatsa, Falkirk Bison Kill, and Menoken Village were placed in both clades. Clade Holocene I also included mtDNA haplotypes from Alkali Creek, Anton Rygh, Bobtail Wolf, Brand Bison Kill, Bundlemaker, Cross Ranch, Double Ditch Village, Larson Village, Shea Farm, Streifel, and White Buffalo Robe. The Taylor Bluff sample was placed in Clade Holocene II (Fig. 2B).

### Diversification of mtDNA haplotypes

We determined that the most active diversification of mtDNA haplotypes in the NGP occurred 12,000–8000 ya during the early Holocene when bison moved to occupy new territory of the retreated ice sheets and drained Lake Agassiz^29^, as grasslands replaced spruce forests. The calibrated Bayesian tree shows 22 nodes of the splitting events in the early Holocene versus 8 during the late Pleistocene–Holocene transition, 7—in the mid Holocene, and 14—in the late Holocene (Supplementary Fig. S5). This result demonstrates that Holocene and modern mtDNA lineages originated in different periods, with most haplotypes appearing in the early Holocene (20 haplotypes, 36.4%) and late Holocene (23 haplotypes, 41.8%) (Supplementary Data S3). Many mtDNA haplotypes that originated in the temporal period from the terminal Pleistocene to the mid Holocene survived thousands of years in the NGP (Supplementary Fig. S5). Interestingly, some mtDNA lineages in modern *B. bison* traced back to their maternal ancestors in the terminal Pleistocene (bHap1 of unknown origin, wHap14 from Elk Island National Park), early Holocene (bHap3, bHap5, bHap7 from a Montana private herd) and mid Holocene (wHap15 from Elk Island National Park). Two lineages of these six belonged to *B. b. athabascae*^28^. The other 6 of 12 modern mtDNA haplotypes (5 from a Montana private herd and 1 from Yellowstone National Park) appeared relatively recently, evolving in the Late Holocene (Supplementary Fig. S5; Supplementary Data S3).

We also confirmed a close relationship between the mtDNA haplotypes of *B. antiquus* and *B. bison* indicated previously through analysis of mtDNA control region^6^. This observation is in agreement with the conclusion that *B. antiquus* did not become extinct but instead evolved to *B. bison* through phenotypic and morphological adaptation to changing environments^7^. Our data also corroborate a close relationship of Holocene mtDNA lineages from the NGP with mtDNA from modern samples and *B. priscus* from the northmost part (Alberta) of the Great Plains.

### Demographic analysis

**Figure 3:**
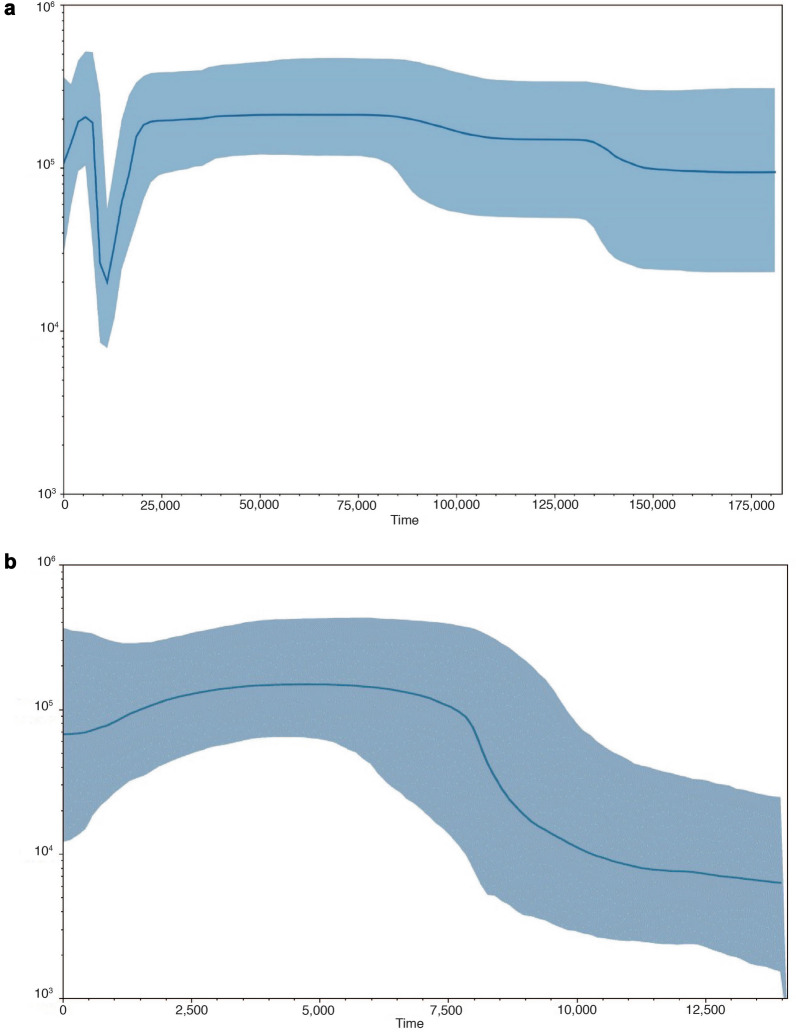
Bison population dynamics. (**a**) Bayesian skyline plot of the N_e_τ dynamics (the y axis) throughout 180,000 years (the x axis) derived from the bison mitogenome sequences of 40 Holocene bison from the NGP (this study), 20 *B. priscus*^4,27^, and 12 bison representing primary modern mtDNA haplotypes^28^. (**b**) Bayesian skyline plot of the N_e_τ dynamics (the y axis) throughout 14,000 years (the x axis) derived from the bison mitogenome sequences of 40 Holocene bison from the NGP (this study). The blue solid line shows the median estimate with the 95% highest posterior density as the shaded area. N_e_, an effective population size based on mtDNA corresponding to female effective population size; τ, a generation time equals to 8 years^30^.

Demographic analysis based on the Late Pleistocene-Holocene-modern set of mitogenomes revealed that long-term population stability occurred from 180,000 ya through 25,000 ya with an N_e_ of 24,376 (95% credibility interval of 11,233–48,188) before the decline and the maximum N_e_ of 26,548 (15,081–58,244) ~ 57,000 ya. A sharp population decline began ~ 20,000 ya during the LGM, dropping almost tenfold to N_e_ of 2495 (985–7031) around 10,000–12,000 ya. This period marked the extinction of many megafaunal taxa in North America, including *B. priscus* in the Arctic. After 10,000 ya, the population began to rapidly grow, reaching the pre-LGM level with N_e_ of 25,709 (12,954–64,979) in the mid Holocene (5500 ya). We then detected a slow decline of bison population beginning in the late Holocene approximately 4000 ya through recent times when N_e_ fell to 13,355 (3726–45,151) (Fig. 3A).

In contrast, demographic analysis of NGP bison in the Holocene based on mitogenome sequences from 40 specimens with the temporal range of 12,226–167 years before present (yr BP) revealed that population dynamics were distinct in several points. This bison population began its origin near the retreating Laurentide ice sheet that covered northern and eastern North Dakota and eastern South Dakota as recently as 12,300 ya^26^ (Fig. 1) and collapsed in the nineteenth century due to the anthropogenic bottleneck. We detected three primary dynamic periods for Northern Plains bison. First, population growth from the small N_e_ of 791 (193–3101) began 14,000 ya with melting the ice sheet and the replacement of spruce forests by the grassland after the LGM and the colonization of new territories that became available after the retreating ice sheet and continued through part of the mid Holocene. Then, demographic stability occurred from 6700 to 2700 ya with N_e_ ranging from 16,414 (3009–52,106) to 18,700 (8043–53,302). Finally, populations fell to N_e_ = 8480 (1570–44,171) just before the nineteenth century extermination of the species (Fig. 3B).

We also compared side-by-side the finer population dynamics of the global bison population and the NGP bison population in the last 14,000 years (Supplementary Fig. S6). This comparison detected a few principal differences. First, despite the decline of the global population that continued until 11,000 yr BP, the Northern Plains bison demonstrated a slow growth since the beginning of colonization of new territories throughout 11,000 ya. In that point the difference in the N_e_ between the global and NGP populations was minimal, 2887 and 1360, respectively. Second, starting ~ 11,000 ya both populations indicated a stable growth till 5500 yr BP with a higher rate in the global population that can be explained by larger territory with various productivity of different ecosystems. The decline first began in the global population shortly after ~ 5500 yr BP while the NGP population was relatively stable until ~ 2700 ya. Around 1800 ya, the difference between both N_e_s was relatively small with the values of 19,314 and 15,853 in the global and NGP populations, respectively. Both populations were relatively stable before their extermination in the nineteenth century.

## Discussion

Our calibrated Bayesian phylogenetic tree consisting of only 40 Holocene mitogenomes confirmed that the common maternal ancestor of Northern Plains mitochondrial genomes lived in the terminal Pleistocene (14,095–23,230 ya) and the main diversification of mtDNA haplotypes occurred in the early Holocene (Supplementary Fig. S7). These haplotypes, without considering indels, indicated haplotype diversity of 0.994 ± 0.008, pairwise difference of 11.25 ± 5.21, and nucleotide diversity of 0.0007 ± 0.0004 (Supplementary Table S1). Some mtDNA sequences from the same site and temporal periods grouped together such as B59 (12,226 yr BP) and B60 (12,109 yr BP) from Beacon Island, B20 (8109 yr BP) and B68 (8114 yr BP) from Rustad. However, all other clades are composed of sequences representing different sites and temporal periods. This analysis disclosed the continuity of diverse mtDNA lineages through the Holocene and the lack of association of mtDNA sequences with archaeological sites in the Northern Plains. The scenario of bison global population dynamics we infer through the Bayesian skyline analysis, based on the largest set of complete mitochondrial genomes analyzed to date, dramatically differs from the demographic model for Beringia bison^1^ but is similar in some points to population changes revealed in other studies^10,16^. Differences can be attributed to variation in modeling methods, the addition of new Holocene mtDNA sequences in our study, and a larger number of genetic polymorphisms in complete mitochondrial genomes versus control region sequences. Regardless, we detected the population decline that continued in both models from 25,000 to 23,000 ya until 12,000–10,000 ya achieving a similar N_e_. After the bottleneck at the terminal Pleistocene, the next wave of population growth and decline happened, indicating another difference in both our and^16^ models. In contrast to^16^, we revealed population growth matching pre-LGM levels in the mid Holocene 5000 ya (Fig. 3A), which could be explained by the better survival of bison and more sustainable environments in the Great Plains than in parts of eastern Beringia, including Interior Alaska and Yukon.

Interestingly, the close values of N_e_ we received for the late Pleistocene and the early–mid Holocene may indicate similar vegetative productivity for Pleistocene steppe-tundra ecosystem in Beringia and the early–mid Holocene Great Plains that were able to support large bison populations. Although, the direct comparison of the productivity of both ecosystems is unknown to us, modern analogues of the Pleistocene steppe-tundra have been identified in the Altai Mountains in southern Siberia and true steppes in Central Asia where other large ungulates persist today^31,32^.

Here, we uncovered that the demographic dynamics of the Northern Plains bison was distinct from both the Beringia population^1,10,16^ and the late Pleistocene–Holocene—modern global population (this study). One distinction was a two-fold population increase in the NGP immediately following the LGM in contrast to continued population decline in both the Beringian population and the global population. Three parts of the population curve demonstrated a strong correlation with environmental and anthropogenic factors that acted in the Northern Plains after the LGM. The original period of population growth correlated with bison colonization of new lands released by melting ice sheets and the development of grasslands at the beginning of the Holocene (12,000 ya). This original population growth accompanied by the adaptation of bison to climatic and environmental changes is thought to be expressed in the decline in bison body size and mass from 12,500 to 9250 ya^7^. The initial entrance of Paleo-Indians into the territory of present North Dakota 11,500 ya^33^ or 9500 ya^34^ did not reverse or delay bison population growth.

The bison population in the Northern Plains in the mid and late Holocene had been stable for at least 4000 years (6700–2700 ya) despite climate fluctuations leading to periodic droughts. The longest drought period known as the Altithermal dry and warm period occurred 8000/7000–5000/4000 ya^33,34^. The stability of bison population during this period can be explained by ecological plasticity and adaptation to heterogeneous landscapes in warmer and drier local climate as well as to the human de-population of the NGP^19,33,35^. In contrast to popular opinion, our analysis indicates that American bison were not at their peak in the Great Plains when the Europeans first arrived in the sixteenth century^14,36^. Instead, we revealed relatively small but continuous population decline starting 2700 ya. The beginning of mass harvesting of bison 2800 ya based on the application of fire and new weapons and hunting technologies in the late Plains Archaic cultural period^13,33,34,37^ is the most parsimonious reason correlating with this decline in the late Holocene. Although mass hunting affected the abundance of bison, it did not result in extirpation, as experienced later in the nineteenth century due to the colonization and industrialization of the Midwest.

Our study has revealed considerable diversity of mtDNA lineages during the Holocene. Despite close phylogenetic association, we found no historic mitochondrial genomes matching modern bison^28^. The scope of lost genetic diversity of the Holocene bison during the anthropogenic bottleneck remains unclear as well as the proportion of the survived Holocene lineages. However, considering the complete extermination of bison in the NGP, 100% of these mtDNA lineages were erased in this geographic population and not found in the modern dataset^28^.

Our research has highlighted the importance of historic and geographical populations as indicators of bison community ecology and adaptation to changing environments. Genetic and isotopic evaluation of diverse temporal and spatial bison populations that occupied North America through the Holocene would benefit our understanding of the species in a natural context and enable development of comprehensive strategies to restore bison herds in the future. To best enable bison success, efforts should focus on identifying prairie territory having productivity comparable to the early–mid Holocene Plains and mammoth steppe-tundra.

## Materials and methods

### Collecting samples

The methodology of sampling bison bone and tooth samples from the collections of North Dakota Heritage Center and State Museum (State Historical Society of North Dakota; permit was provided by Fern Swenson, Director of the Archaeology and Historic Preservation Division), Knife River Indian Villages National Historic Site (National Park Service permit KNRI-2015-SCI-0006), and University of North Dakota’s Department of Anthropology (permit was provided by Dennis Toom, Research Archaeologist Emeritus of Anthropology) was described earlier^19^. We cut or drilled a section of teeth or bones for ancient DNA analysis before sampling tissues for radiocarbon dating and stable isotope profiling^19^. Information about archaeological sites, types of specimens, and calibrated age in yr BP was included in Supplementary Data S1. Examples of bison samples are shown in Supplementary Figs. S1 and S2.

### DNA isolation

The project was carried out in a laboratory designated for ancient DNA work at the University of North Dakota. All samples were cleaned by removing soil and dirt deposits using sandpaper. Specimen surfaces were de-contaminated by consequently washing in or wiping with a bleach solution (2% sodium hypochlorite), molecular biology grade water and ethanol. Finally, samples were dried under UV irradiation. All de-contamination steps were conducted within a biosafety cabinet assigned for processing ancient bison materials. We sampled bone and dentin powder samples using a drill with hole saw bits at low drill speeds as recommended elsewhere^38^. We performed DNA isolation from 268 to 370 mg of bone or tooth powders using a DNA extraction method optimized for low-quality samples^39^. Concentrations of DNA isolated from samples were measured by Qubit fluorometry using a high sensitivity assay for low DNA concentrations (Fisher Scientific) (Supplementary Data S1).

### Library preparation

We prepared dual-indexed DNA libraries from single-stranded DNA (ssDNA) using the Accel-NGS Methyl Seq DNA library kit with an uracil-tolerant polymerase (Swift Biosciences) according to the manufacturer’s protocol for the retention of small DNA fragments. The bisulfite conversion step included in the kit was not used to obtain DNA libraries. The Accel-NGS Methyl Seq DNA library technology adds a low complexity polynucleotide tail with an average length of 8 bases to the 3ʹ end of each fragment and may also add small tails to the 5ʹ ends during end repair steps (Swift Biosciences Technical Note). Depending on the input amount of DNA, the variable numbers of PCR cycles were used to generate DNA libraries according to the manufacturer’s protocol. Retention of small DNA fragments was regulated by different ratios of magnetic SPRI beads (Beckman Coulter) in solutions during purification steps, as recommended by Beckman Coulter and Swift Biosciences. Concentrations of DNA libraries were measured by Qubit fluorometry using a high sensitivity assay for low DNA concentrations (Fisher Scientific) (Supplementary Data S1).

The DNA libraries were captured by Daicel Arbor Biosciences (https://arborbiosci.com/) using a myBaits Expert Mito kit targeting the full *B. bison* mitochondrial genome (NC_012346). One round of hybridization capture was performed using 4-plex reactions following the myBaits manual (version 4.01) with hybridization and washes steps at 60 °C. Following capture, the first 24 samples were amplified for 10 cycles and the second 23 samples were amplified for 14 cycles. The DNA libraries were sequenced using the Illumina MiSeq platform with SE 150 (150 bp single-end) chemistry. The DNA libraries were sequenced at the University of North Dakota’s Genomics Core (24 libraries) and Daicel Arbor Biosciences (23 libraries).

### Estimation of consensus mitochondrial genome sequences and summary population statistics

To produce consensus sequences of the mitochondrial genomes, we tested two approaches. First, DNA reads were processed with BBDuck (http://sourceforge.net/projects/bbmap/) incorporated into Geneious Prime 2021.1.1 (https://www.geneious.com) with default settings for trimming the Illumina adapters and low complexity sequences added to the 3ʹ and 5ʹ ends, together with low quality trimming and removing short (< 20 bp) and duplicate reads. DNA reads were mapped using Geneious Prime with default parameters for a modern bison mitochondrial genome (GU946990) sequenced from an animal from a private herd in Montana^28^. To produce consensus mitogenome sequences, we used Geneious Prime with consensus threshold set at the highest quality of 95%.

Second, because BBDuck does not include the option of removing PCR duplicates, we processed the raw DNA reads and the DNA reads mapped to GU946990 using Paleomix^40^. Short reads with length less than 25 bp were discarded with AdapterRemoval. Picard MarkDuplicates tools v2.26 incorporated in this pipeline were set to “filter” to remove PCR duplicates. For consensus SNP calling, we selected the depth of coverage equals 3^40^.

In total, we obtained 40,860,967 raw DNA reads. The result of reads' processing in Geneious and Paleomix is shown in Supplementary Data S1. The estimated mean coverage ranged from 24 to 7775 in Geneious and from 19 to 299 in Paleomix. The total number of reads mapped to GU946990 ranged from 2818 to 930,448 in Geneious and from 2434 to 32,734 in Paleomix. More robust data received in Paleomix were used for final consensus base calling.

To identify single nucleotide polymorphisms (SNPs), the consensus mitochondrial genome sequences obtained in this study were aligned with the reference bison mitogenome (GU946990) using MEGA7^41^. SNPs detected in the Holocene bison mitogenomes are shown in Supplementary Data S2. Population diversity statistics of 40 Holocene bison (this study) as well as 19 Pleistocene *B. priscus*^4^ and 32 modern bison^28^ for comparison was determined using ARLEQUIN ver. 3.5.2.2^42^ after removing gaps (insertions, deletions) and positions with undetermined (N) nucleotides (Supplementary Table S1).

### The estimation of DNA damage parameters

Ancient DNA damage parameters of single-end DNA reads were analyzed by mapDamage 2.0^43^. In ancient DNA molecules, it is expected that single-end DNA reads have an increased frequency of C to T misincorporations at the 5ʹ ends as the consequence of cytosine deamination (Supplementary Fig. S3) and higher frequency of guanine or adenine in the − 1 position of DNA breaking points in front of the 5ʹ end of DNA reads (Supplementary Fig. S4)^44^.

### Phylogenetic analyses

To understand the evolutionary relationships of 40 Holocene bison mitogenome sequences obtained in this study, we selected the best 19 mitochondrial genome sequences of *B. priscus* from^4^ and one—from^27^ isolated from dated remains and having no stretches of undetermined (N) nucleotides. We also included 12 modern bison mitogenome sequences representing the main modern haplogroups^28^.

We have excluded from consideration partial mitogenome sequences with missing parts from Holocene steppe bison from the Yukon^21^ and from two historic bison remains sampled in northern Wyoming in 1856 and southern Montana in 1886^45^.

### Bayesian phylogenetic analysis of mtDNA sequences with MrBayes

We utilized MrBayes 3.2.6^46^ implemented in Geneious Prime to estimate a Bayesian phylogenetic tree using the HKY + I + G substitutional model as determined by jModelTest2^47^. We ran the Markov chain Monte-Carlo (MCMC) analysis using 1,100,000 generations with 4 heated chains, a burn-in length of 100,000, and sampling every 200 generations. The MCMC convergence was diagnosed by the average standard deviation of split frequencies (ASDSF)^46^ and the trace plots in Tracer^48^. Phylogenetic trees were visualized using FigTree 1.4.4 (http://tree.bio.ed.ac.uk/software/figtree/).

### Bayesian phylogenetic analysis of mtDNA sequences with the median calibrated radiocarbon dates used to calibrate the tree

We analyzed two datasets of bison mtDNA sequences. The first set included complete mitochondrial genomes from 40 Holocene bison (this study), 20 Pleistocene *B. priscus* (19 sequences from^4^ and one sequence from^27^) and 12 modern bison lineages^28^. The second set contained only 40 Holocene bison mitogenome sequences obtained in this study.

We used BEAST 1.10.4^49^ to build Bayesian phylogenetic trees based on complete mitochondrial DNA sequences and their radiocarbon age using the HKY + I + G substitution model for the late Pleistocene–Holocene–modern set and the HKY + G substitution model for the Holocene set according to the jModelTest 2 definition^47^, a strict molecular clock, and the Bayesian Skyline coalescence model with ten groups. The MCMC analysis ran 10,000,000 generations with sampling each 1000 generations with the convergence diagnosed in Tracer^48^.

TreeAnnotator (http://beast.bio.ed.ac.uk) was used to make calibrated phylogenetic trees. TreeAnnotator in BEAST 1.10.4 package^49^ was used to compile the sampled trees in a single target tree to annotate summarized information. Phylogenetic trees were visualized using FigTree 1.4.4 (http://tree.bio.ed.ac.uk/software/figtree/).

### Demographic analysis

We analyzed two datasets of bison mtDNA sequences described above using the Bayesian Skyline model previously applied to steppe bison demographic studies^16,50,51^. To reveal bison population demography over time, we analyzed log files generated in BEAST 1.10.4 using Tracer 1.7.1^48^. Tracer produced graphical plots of demographic reconstructions showing changes of N_e_τ (the y axis), where N_e_ is an effective population size based on mtDNA corresponding to female effective population size and τ is a generation time, over time in radiocarbon years before the present (the x axis)^16^. We selected 8 years as the bison generation time in this study^30^ although bison generation time has ranged 3–10 years by different authors^7^.

## Supplementary Information

Supplementary Information 1.

Supplementary Information 2.

Supplementary Information 3.

Supplementary Information 4.

## Data Availability

All data are available in the main text or the Supplementary materials. Original fastq files with accession number PRJEB62763 are deposited in the European Nucleotide Archive (ENA).
