# Supplementary material for: Mitogenomes revealed the history of bison colonization of Northern Plains after the Last Glacial Maximum
Source: Sci Rep. 2023 Jul 14;13:11417. doi: 10.1038/s41598-023-37599-8 (PMC10349043; doi:10.1038/s41598-023-37599-8)
Supplement: Supplementary file 4 — Supplementary Information 4. [file 41598_2023_37599_MOESM4_ESM.docx]

**Supplementary Information**

**Mitogenomes revealed the history of bison colonization of Northern Plains after the Last Glacial Maximum**

Igor V. Ovchinnikov^1*^, Blake McCann^2^

^1^Department of Biology, University of North Dakota, Grand Forks, ND, USA

^2^Theodore Roosevelt National Park, Medora, ND, USA

*Corresponding author. Email: igor.ovtchinnikov@und.edu

**Supplementary figures**


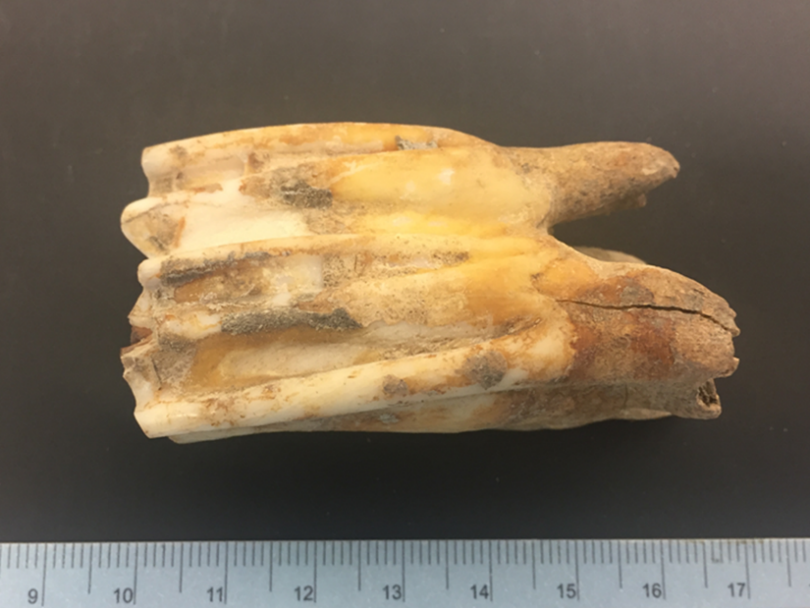


**Supplementary Figure S1.** An example of bison tooth used for DNA analysis; Bison 48, Rustad, 7,916 yr BP.


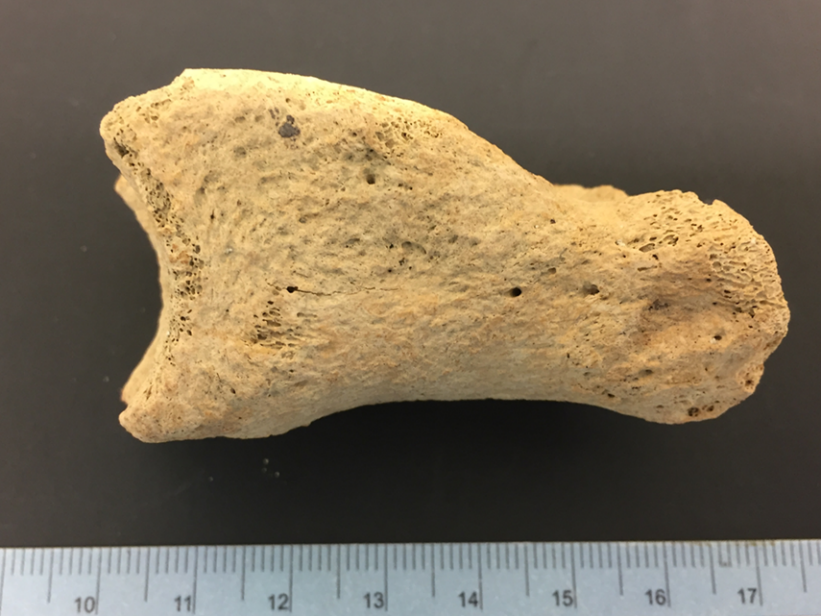


Supplementary Figure S2. An example of bison bone used for DNA analysis; Bison 64, phalanx, Mondrian Tree, 3,733 yr BP.


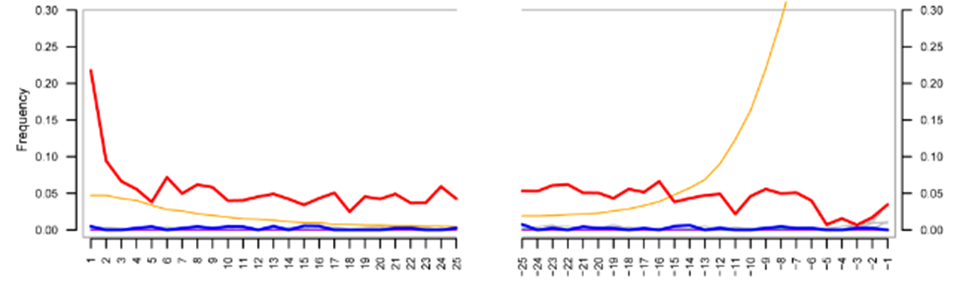


**Supplementary Figure S3.** Typical plots generated by mapDamage 2.0 (library made from DNA isolated from B66, Rustad, 8,116 yr BP) showing frequencies of C to T misincorporation caused by deamination of C leading to increased frequency of thymine (the red line) at the 5’ ends of ancient single-end DNA reads distinguishing them from modern DNA contamination (44, 52). The blue line shows the frequencies of adenines. The orange line indicates soft-clipped bases belonging to low complexity tails added to the 3’ and 5’ ends of DNA fragments in the adaptase step of the Accel-NGS Methyl-Seq technology (Swift Biosciences). To demonstrate that ancient DNA damage patters are not obscured, we have not trimmed the low complexity tails in this example.


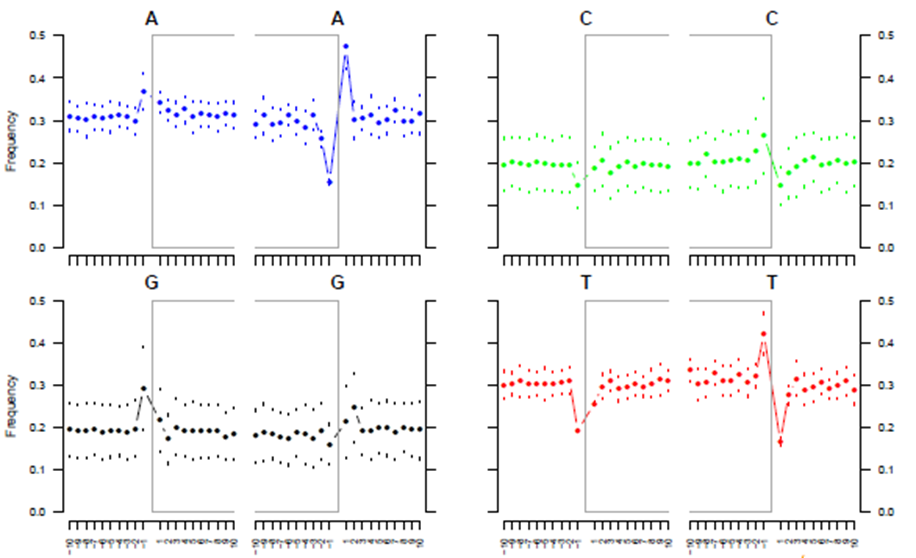


**Supplementary Figure S4.** Typical distribution of nucleotide frequencies around DNA strand breaks (shown by the gray brackets) generated by mapDamage 2.0. Purines (A and G) indicate an increased frequency at 5’ position -1 of single-end ancient DNA reads (44, 52).


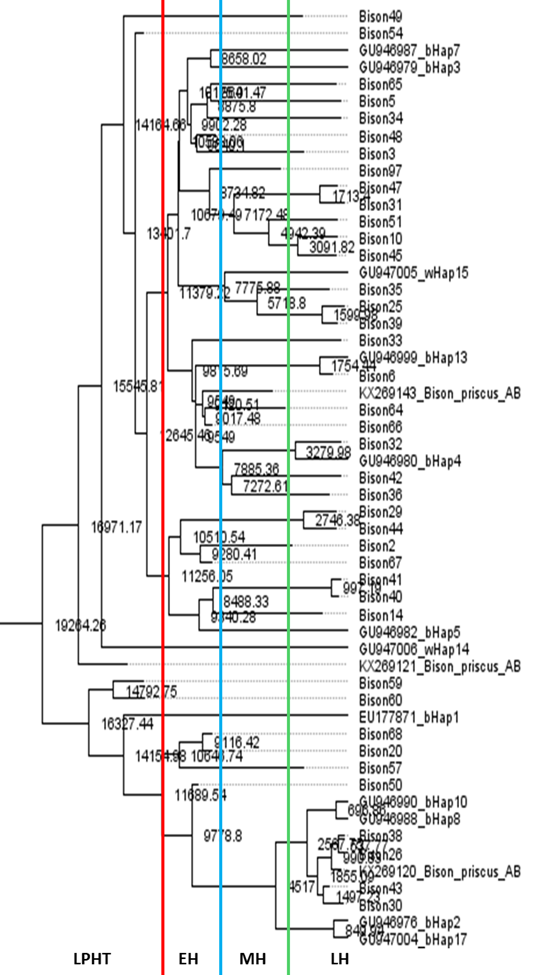


Supplementary Figure S5. Diversification of mtDNA haplotypes after the LGM. The detailed Bayesian phylogenetic tree of the mitochondrial genome sequences calibrated according to the ages of bison remains. The tree includes the mtDNA sequences of 40 Holocene bison from the NGP obtained in this study, 3 *B. priscus* (4) incorporated into the Holocene dataset and 12 modern bison representing the main modern mtDNA haplotypes (28). Node numbers refer to calibrated years before present. LPHT, late Pleistocene – Holocene transition; EH, early Holocene; MH, mid Holocene; LH, late Holocene (53).


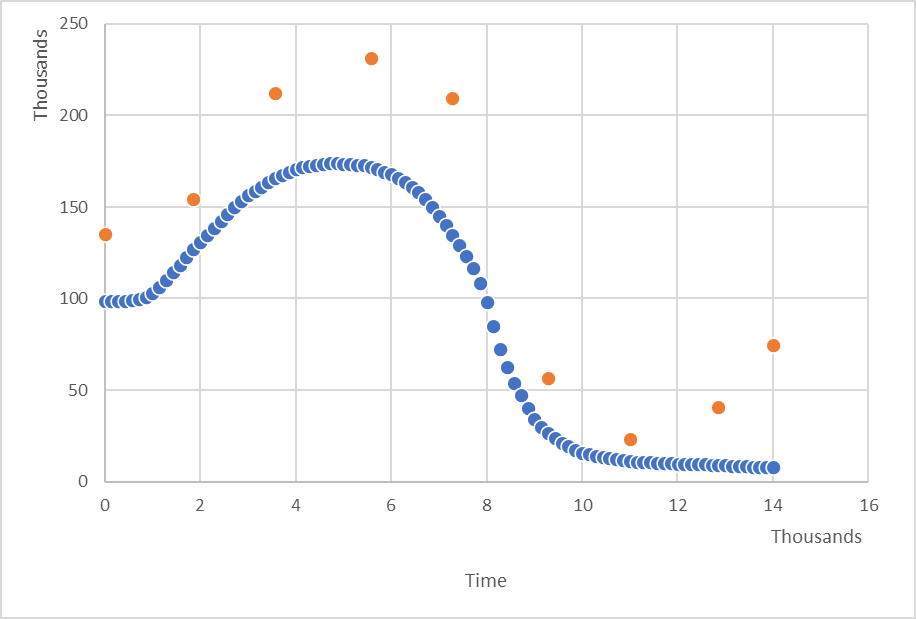


**Supplementary Figure S6.** The detailed comparative reconstructions of the 14,000-year-old population dynamics of the NGP bison based on 40 mitogenomes (this study; blue dots) and the global bison population consisting of the mitochondrial genomes of 40 Holocene bison (this study), 20 *B. priscus* (4, 27), and 12 bison representing primary modern mtDNA haplotypes (28) (orange dots). The y axis shows the N_e_τ dynamics throughout 14,000 years (the x axis).


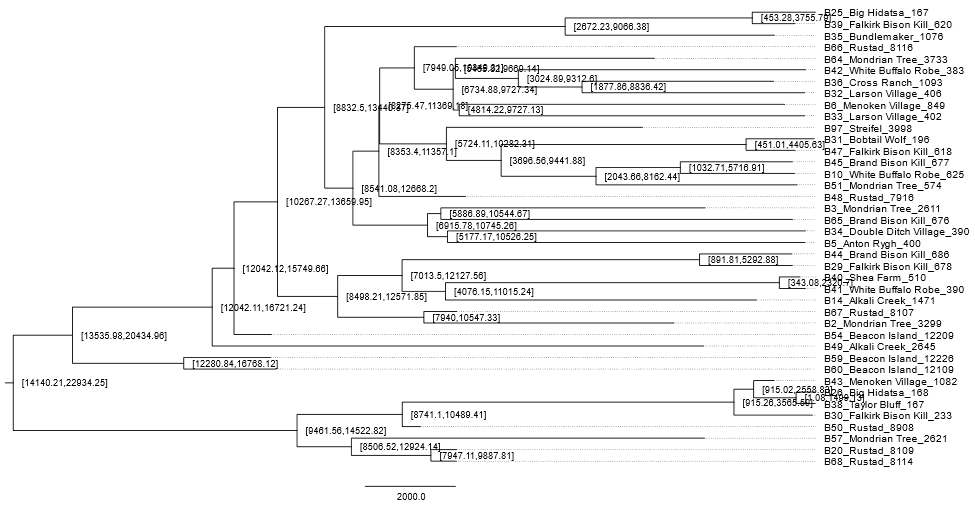


**Supplementary Figure S7.** Bayesian phylogenetic tree of the mitochondrial genome sequences of 40 Holocene bison from the NGP calibrated according to the ages of bison remains. Tip labels indicate bison specimen, archaeological site, and calibrated age in yr BP. Node ranges show 95% confidence intervals of ages in yr BP.

**Supplementary table**

**Supplementary Table S1:** Summary population statistics in Holocene, Pleistocene and modern bison based on the complete mitochondrial genome sequences (indels are not counted with del weight = 0).

| Temporal population | # of samples | # of haplotypes  (without considering indels) | Ts / Tv | Haplotype diversity | Mean pairwise difference | Nucleotide diversity | Reference for mtDNA |
| --- | --- | --- | --- | --- | --- | --- | --- |
| Holocene | 40 | 36 | 98 / 7 | 0.9936 ± 0.00770 | 11.2474 ± 5.2141 | 0.000690 ± 0.000355 | This study |
| Pleistocene | 19 | 18 | 340 / 12 | 0.9942 ± 0.0193 | 72.4035 ± 32.6513 | 0.004457 ± 0.002246 | 4 |
| Modern | 32 | 17 | 63 / 8 | 0.9012 ± 0.0462 | 15.6532 ± 7.1743 | 0.000959 ± 0.000489 | 28, 54 |

**Supplementary references**

52. Matisoo-Smith, E.A. et al. A European mitochondrial haplotype identified in ancient Phoenician remains from Carthage, North Africa. *PLoS One* **11**, e0155046 (2016).

53. Walker, M.J.C. et al. Formal subdivision of the Holocene Series/Epoch: A discussion paper by a Working Group of INTIMATE (Integration of ice-core, marine and terrestrial records) and the Subcommission on Quaternary Stratigraphy (International Commission on Stratigraphy). *J. Quat. Sci.* **27**, 649-659 (2012).

54. Achilli, A. et al. Mitochondrial genomes of extinct aurochs survive in domestic cattle. *Curr. Biol.* **18**, R157-R158 (2008).

Supplementary Data files

Supplementary Data S1. Characteristics of bison specimens and DNA libraries used to generate complete mitochondrial genome sequences.

Supplementary Data S2. Single nucleotide polymorphisms (SNPs) in 40 Holocene bison mitochondrial genomes aligned to GU946990.

Supplementary Data S3. Chronological origin of sample-specific mtDNA haplotypes found in Holocene, modern and latest *B. priscus* specimens.
